# Ferric carboxymaltose with or without erythropoietin for the prevention of red-cell transfusions in the perioperative period of osteoporotic hip fractures: a randomized contolled trial. The PAHFRAC-01 project

**DOI:** 10.1186/1471-2474-13-27

**Published:** 2012-02-21

**Authors:** Máximo Bernabeu-Wittel, Reyes Aparicio, Manuel Romero, José Murcia-Zaragoza, Rafael Monte-Secades, Clara Rosso, Abelardo Montero, Alberto Ruiz-Cantero, María Melero-Bascones

**Affiliations:** 1 Hospital Universitario Virgen del Rocío, Sevilla, Spain; 2 Hospital San Juan de Dios del Aljarafe, Sevilla, Spain; 3 Hospital Infanta Elena, Huelva, Spain; 4 Hospital de la Vega Baja, Orihuela, Alicante, Spain; 5 Complexo Hospitalario Xeral-Calde, Lugo, Spain; 6 Hospital Universitario de Bellvitge, Barcelona, Spain; 7 Hospital de la Serranía, Ronda, Málaga, Spain; 8 Hospital General Universitario de Albacete, Albacete, Spain; 9 Department of Internal Medicine, Hospitales Universitarios Virgen del Rocío, Avenida Manuel Siurot s/n., 41013 Sevilla, Spain

**Keywords:** Hip fracture, Transfusion, Blood-saving strategies, Ferric carboxymaltose, Erythropoietin, Red-cell pack, Clinical trial

## Abstract

**Background:**

Around one third to one half of patients with hip fractures require red-cell pack transfusion. The increasing incidence of hip fracture has also raised the need for this scarce resource. Additionally, red-cell pack transfusions are not without complications which may involve excessive morbidity and mortality. This makes it necessary to develop blood-saving strategies. Our objective was to assess safety, efficacy, and cost-effictveness of combined treatment of i.v. ferric carboxymaltose and erythropoietin (EPOFE arm) versus i.v. ferric carboxymaltose (FE arm) versus a placebo (PLACEBO arm) in reducing the percentage of patients who receive blood transfusions, as well as mortality in the perioperative period of hip fracture intervention.

**Methods/Design:**

Multicentric, phase III, randomized, controlled, double blinded, parallel groups clinical trial. Patients > 65 years admitted to hospital with a hip fracture will be eligible to participate. Patients will be treated with either a single dosage of i.v. ferric carboxymaltose of 1 g and subcutaneous erythropoietin (40.000 IU), or i.v. ferric carboxymaltose and subcutaneous placebo, or i.v. placebo and subcutaneous placebo. Follow-up will be performed until 60 days after discharge, assessing transfusion needs, morbidity, mortality, safety, costs, and health-related quality of life. Intention to treat, as well as per protocol, and incremental cost-effectiveness analysis will be performed. The number of recruited patients per arm is set at 102, a total of 306 patients.

**Discussion:**

We think that this trial will contribute to the knowledge about the safety and efficacy of ferric carboxymaltose with/without erythropoietin in preventing red-cell pack transfusions in patients with hip fracture. ClinicalTrials.gov identifier: NCT01154491.

## Background

Hip fracture (HF) is highly prevalent in Spain, mainly affecting the elderly. It is estimated that 0.7% of women 65 years or older will suffer a hip fracture during their lifetime, and demographic trends predict that the demand for care will double in the next 40 years [1-3]. A total of 4-8% of these patients will die during hospitalization, and fewer than 25% will regain mobility and preserve autonomy at hospital discharge [4,5]. Socioeconomic burden associated to HF is enormous (40.000 euros (57.000 US dollars)) per patient [6].

Anemia is the one of the most frequent complications of HF; around one third to one half of patients will require red-cell pack(s) (RCP) transfusion [7]. RCP transfusion has been the classical choice in treating severe anemia of these patients [7]. Nevertheless, as already stated, the increasing incidence of HF in this population has also raised the need for this valuable and scarce resource. Additionally, RCP transfusions are not without complications [8-11]. In very old and often fragile patients, these effects may involve excess morbidity and mortality, making it necessary to develop blood-saving strategies (BSS) [9,12].

The most important factor in the development of anemia is blood loss, but some other mechanisms (renal failure, inflammation, iatrogenic hemodilution) are also implicated, prompting the choice of different therapeutic approaches [9,13,14]. These rely in three cornerstones: firstly minimizing blood loss in the operating room, secondly rationalizing RCP transfusion protocols, and thirdly enhancing bone-marrow red blood cell production [13-16]. Although evidence that this last measure saves RCP is weak, an increasing number of works have demonstrated notable reductions in RPC transfusion, nosocomial infections, hospital stay, and even survival [14-16]. Nevertheless, some biases in the nature of said studies could partly explain some of the obtained results.

All these previous studies were performed using 300 to 600 mg of intravenous iron sucrose administered in a period ranging from 2 to 6 days. Ferric carboxymaltose (FC) has considerable advantages over iron sucrose because it allows doses of 1 g in a single session, equivalent to 1000 mg of iron sucrose. Moreover, FC also provides an excellent tolerability and safety profile [17-19]. Therefore, it is very useful in situations where quick replacement is required as is the case of HF. It has been used successfully in iron deficiency-, inflammatory-, and in postpartum anemia, however there are no data on its efficacy in HF.

Preliminary results of perioperative use of EPO in HF are promising; however, these results suffer from bias because trials were not randomized and/or blinded. On the other hand, all studies were performed with iron sucrose, so that any additional benefit related to FC cannot be extrapolated. Therefore we have promoted and developed a phase III clinical trial (CT) in order to definitively assess the benefit of RCP saving with the use of EPO with/without FC in patients with HF. The main objective of the present CT was to evaluate the efficacy of combined treatment of i.v. ferric carboxymaltose and erythropoietin (EPOFE arm) versus i.v. ferric carboxymaltose (FE arm) versus placebo (placebo arm) in reducing the percentage of patients who receive blood transfusions in the perioperative period of hip fracture intervention.

## Methods

This is a multicentric, randomized, controlled, double blinded clinical trial of parallel groups, performed on adult in-hospital patients admitted with an HF. The eligible patients will be those admitted to the Emergency-Traumatology Services from the moment of admission until the moment of surgery. Inclusion and exclusion criteria are detailed in Table 1. Once the patient is eligible, meets inclusion criteria, does not meet any exclusion criteria and signs the informed consent form to participate in the clinical trial, the investigator will proceed to randomize the patient to the intervention group, and to perform the analytical extraction of blood count prior to the administration of the first dose of the trial drugs. The investigators will be responsible for having provided appropriate information about objectives, methods, anticipated benefits and potential risks to all eligible patients. If the patient is unable to read or to give consent, the legal representative must be present during the informed consent process and the legal representative will sign the consent form to attest that the information contained in the form has been explained and understood accurately and, where appropriate, to give consent. The investigator must also explain that patients are completely free to refuse to participate in the study or withdraw at any time for any reason.

**Table 1:** Inclusion and exclusion criteria for entering the PAHFRAC-01 clinical trial

INCLUSION CRITERIA	EXCLUSION CRITERIA
Age ≥65 years	Bone marrow diseases which could interfere in the erythropoietic process (acute or chronic myelodysplastic syndromes or myeloproliferative diseases, and/or infiltration of the bone marrow due to solid or lymphatic neoplasm)

Osteoporotic hip fracture requiring surgical repair	Blood coagulation diseases or being currently treated with oral anticoagulants and/or heparin at therapeutic doses.

Hemoglobin levels between 90-120 g/L	Documented allergy and/or previous intolerance and/or contraindication of erythropoietin use and/or intravenous iron

Signed informed consent form	Patients with rheumatoid arthritis and/or another demonstrated origin of inflammatory anemia and/or not controlled arterial hypertension
	
	Patients with current or previous treatment for at least 3 months, with erythropoietin or intravenous iron
	
	Patients with chronic renal failure receiving hemodialysis or peritoneal dialysis

A randomization assignment list will be stratified by center. The randomization list for each center will be sent to the hospital Pharmacy Service at the time of the drug delivery. The person responsible for clinical trials in the Pharmacy Service will be the one responsible for keeping the blinded assignment. The allocation and blinding will be done by masking the syringes of EPO/placebo and iron infusion in the Pharmacy Service of each of the participating centers. The highest quality standards for handling parenteral medications will be kept for the preparation of intravenous mixtures. FC will be performed using plastic bags and opaque infusion sets.

Treatment will be administered in the three arms detailed in Table 2: Erythropoietin + Intravenous ferric carboxymaltose (EPOFE ARM); Placebo of Erythropoietin + Intravenous ferric carboxymaltose (FE ARM); Placebo of Erythropoietin + placebo of intravenous ferric carboxymaltose (PLACEBO ARM). The trial treatment will be administered, regardless of the arm, as soon as possible after inclusion, and always before surgery. Patients will receive the best standard treatment based on protocols existing in each hospital and the best medical care from both the internal medicine and orthopedic teams. Neither intravenous iron nor erythropoietin will be administered to the patient beyond that provided within the clinical trial. All patients could receive complementary oral iron if it is clinically indicated. Additionally, in clinical practice if the hemoglobin at discharge is less than 120 g/L, and no contraindications exits, oral iron regimen will be prescribed to the patient. The promotor's center (Hospital Universitario Virgen del Rocío) will provide the drugs for the trial, experimental and control treatments. The labeling and distribution of the study drugs will be managed by the Pharmacy Service of Virgen del Rocío University Hospital after having obtained the Spanish Regulatory approval for these procedures.

**Table 2:** The three treatment arms of the PAHFRAC-01 clinical trial

TREATMENT ARM	TREATMENT DESCRIPTION
EPOFE ARM	Single dose of 40.000 IU of erythropoietin in a pre-filled 1 ml syringe, subcutaneously. Ferric carboxymaltose 1000 mg (two 500 mg vials diluted in a bottle of 250 ml of saline, in a plastic bag, and with an opaque infusion system), intravenously in a 20-min infusion.

FE ARM	Single dose placebo (saline) in a pre-filled 1 ml syringe, subcutaneously. Ferric carboxymaltose 1000 mg (two 500 mg vials diluted in a bottle of 250 ml of saline, in a plastic bag, and with an opaque infusion system), intravenously in a 20-min infusion.

PLACEBO ARM	Single dose placebo (saline) in a pre-filled 1 ml syringe, subcutaneously. Ferric carboxymaltose 1000 mg (two 500 mg vials diluted in a bottle of 250 ml of saline, in a plastic bag, and with an opaque infusion system), intravenously in a 20-min infusion.

**Figure 1:**
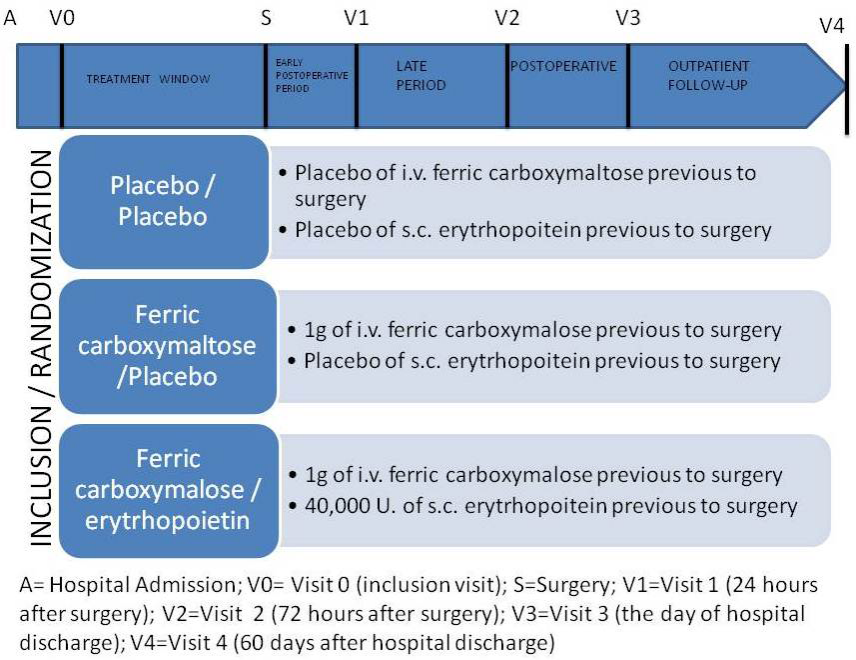
**Scheme of the clinical trial visits in the three arms**. V0 = Visit 0; S = Surgery; V1 = Visit 1 (24 hours after surgery); V2 = Visit 2 (72 hours after surgery); V3 = Visit 3 (the day of hospital discharge); V4 = Visit 4 (60 days after hospital discharge). A = Ferric carboximaltose + Erythropoietin; B = Ferric carboximaltose + Placebo; C = Placebo + Placebo

Patients will be examined by the clinical support team and the Traumatology team at every visit during the study. The data will de classified by visit, and logged in the Clinical Report Data Form (Figure 1).

### Study visits

Clinical, analytical and exploratory data needed for the study will be collected by the investigator. Clinical trial visits are structured in five stages as detailed below:

-Visit 0: Screening day.

-Visit 1: In the first 24 hours postoperative period.

-Visit 2: After the first 72 hours postoperative period.

-Visit 3: The day of hospital discharge.

-Visit 4: 60 days at hospital discharge.

### Withdrawal from the study

Each participant will have the right to withdraw from the study at any time. In addition, the investigator may remove a participant from the study at any time if it is considered necessary for any reason including: significant protocol deviation, significant non-compliance with treatment regimen or study requirements, an adverse event which requires discontinuation of the study medication or results, inability to continue to comply with study procedures, consent withdrawn, and loss of follow up.

All the patients leaving the study will fulfill the described procedures of premature finalization as described in the protocol. The analysis will be carried out for all the patients who have received a dose of experimental medicine or control.

### Outcome measures

The primary outcome measure will be the percentage of patients who receive red-cell transfusion during hospitalization, and after 60 days of hospital discharge.

Secondary outcome measures will be the following related with efficacy, safety, HRQoL, and cost-effectiveness

Efficacy end-points: the average number of red-cell packs needed per patient at the end of the study, the average number of red-cell packs needed per patient (only transfused patients), the average hemoglobin level at 24 and 72 hours postoperative period and at hospital discharge, and the total number of hospitalization days.

Safety endpoints: the death rate from all causes at the end of the study; and adverse events including the following clinical complications: vascular events, all kinds of cerebrovascular accidents, acute coronary syndrome, thrombosis, arterial embolism, heart failure, acute/chronic exacerbation of respiratory failure, delirium, thromboembolic disease, catheter-related phlebitis; renal failure; infectious complications: pneumonia, urinary tract infection, surgical-site infection; and all side effects detailed on the EPO and FC data sheet.

HRQoL end-point: Quality of life will be assessed by the Short form 36 version 2 (SF-36) for acute patients [20]. This questionnaire will be administered at inclusion and 60 days after hospital discharge.

Cost-effectiveness end-point: An analysis of incremental cost-effectiveness (using as a primary endpoint each patient not requiring a transfusion, and as a secondary endpoint every patient who survived the index admission) will be performed.

### Sample size calculation

Sample size was calculated as follows:

-To obtain statistical significance in the comparison between the i.v. iron + EPO arm compared to placebo. The treatment arm exclusively with iron is only used in the evaluation of results, but not in the sample size calculation.

-To obtain a confidence level of 95% and 80% power.

-To be single sided.

-To obtain a reduction in the primary endpoint of 20% (percentage of patients who need transfusions), which is considered as clinically significant in the literature. According to this, we would expect the percentage of patients who need to be transfused in the placebo group to be 60% and in the treatment group 40%.

-Sample size was calculated online using SISA http://www.quantitativeskills.com/sisa/calculations/samsize.htm. A total of 87 patients per arm are needed with the foregoing conditions.

-Taking into account a percentage of drop-outs of 15% per arm, the total number of patients per arm is set at 87 × (1/1-0.15) = 102, a total of 306 patients.

### Statistical analysis

Both intention to treat (ITT) and per protocol analysis will be performed. The ITT analysis will include all patients who agreed to participate in the study, signed the informed consent, and had been randomized. Per protocol analysis will include the patients who were randomized, received the dose of erythropoietin and ferric carboxymaltose (EPOFE arm), ferric carboxymaltose (FE arm), or placebo (PLACEBO arm), and were operated on; excluding those who discontinued the trial, or received a red-cell pack in a different format than specified in the protocol. Violation of the blinded assignment will be also excluded in the per protocol analysis.

A chi-square test will be performed in order to establish the comparative analysis of the primary efficacy outcome, secondary efficacy, safety and cost endpoints between different arms of the study (Yates correction and, where necessary, Fisher exact test) for qualitative endpoints. Analysis of variance (with post-hoc test of Tukey and T3-Dunette)/Kruskal-Wallis test will be performed for quantitative variables. Differences between groups will be quantified using 95% confidence intervals.

The cost-effectiveness analysis will be performed by incremental cost-effectiveness method, using each patient who did not require RCP transfusion as the primary efficacy unit; each patient surviving at discharge and 60 days after discharge as the high efficiency unit; and the sum of all previously recorded costs of hospitalization as the cost unit. The calculations will be performed as follows: incremental cost-effectiveness (Euros per unit of efficiency gains) = NNT × (cost per a) option patient - cost per b) option patient).

All calculations will be performed using SPSS 18.0. The threshold of statistical significance is set at *p *< 0.05.

### Interim analysis and stopping rules

An interim analysis will be performed when half of the patients are recruited, blinded to investigators in order to a) detect possible imbalances between the three arms of the trial with respect to a list of independent secondary endpoints, and b) assess whether the primary efficacy endpoint was obtained at this point in the trial. The list of independent secondary endpoints will include the following: age, sex, type of fracture (subcapital/intertrochanteric), baseline hemoglobin level and type of surgery.

### Adverse events (AE), adverse reactions (AR), and safety issues

#### Definition of AE

Any untoward medical occurrence in a patient or clinical investigation participant administered a medicinal product, which does not necessarily X have a causal relationship with this treatment (the study medication). An AE can therefore be any unfavorable and unintended sign (including an abnormal laboratory finding), symptom or disease temporarily associated with the use of the study medication/procedure, whether or not considered related to the study medication.

#### Definition of AR

All untoward and unintended responses to a medicinal product related to any dosage. "Responses to a medicinal product" means that a causal relationship between a study medication and an AE is at least a reasonable possibility, i.e., the relationship cannot be ruled out. All cases judged by either the reporting medically qualified professional or the sponsor as having a reasonable suspected causal relationship to the study medication/procedure qualify as adverse reactions.

#### Definition of Serious Adverse Event (SAE)

A serious adverse event is any untoward medical occurrence that at any dosage, results in: death, any life-threatening event, any event requiring hospitalization or prolongation of actual hospitalization, any event resulting in persistent or significant disability/incapacity, the occurrence of a congenital anomaly/birth defect, or other important medical event (other events that may not result in death are not life threatening, or do not require hospitalization, may be considered a serious adverse event when, based upon appropriate medical judgment, the event may jeopardize the patient and may require medical or surgical intervention to prevent one of the outcomes listed above.

To ensure no confusion or misunderstanding of the difference between the terms "serious" and "severe", which are not synonymous, the following note of clarification is provided

The term "severe" is often used to describe the intensity (severity) of a specific event (as in mild, moderate, or severe myocardial infarction); the event itself, however, may be of relatively minor medical significance (such as severe headache).

#### Definition of Serious Adverse Reaction (SAR)

An adverse event (expected or unexpected) that is both serious and, in the opinion of the reporting investigator, believed with reasonable probability to be due to one of the study treatments, based on the information provided.

#### Definition of Suspected Unexpected Serious Adverse Reaction (SUSAR)

A serious adverse reaction, the nature or severity of which is not consistent with the applicable product information (e.g. Investigator's Brochure for an unapproved investigational product or summary of product characteristics for an approved product).

#### Causality and expectedness

The relationship of each adverse event to the trial medication will be determined by a medically qualified clinician according to the following definitions

#### Related

The adverse event follows a reasonable temporal sequence from trial medication administration. It cannot reasonably be attributed to any other cause.

#### Not related

The adverse event is probably produced by the participant's clinical state or by other modes of therapy administered to the participant.

#### Procedures for recording AE

All AEs occurring during the study/or until observed by the investigator or reported by the participant, whether or not attributed to study medication, will be recorded on the CRF. The following information will be recorded: description, date of onset and end date, severity, assessment of relatedness to study medication, other suspect drug or device and action taken. Follow-up information should be provided as necessary.

AEs considered related to the study medication as judged by a medically qualified investigator or the sponsor will be followed until resolution or the event is considered stable. All related AEs that result in a participant's withdrawal from the study or are present at the end of the study, should be followed up until a satisfactory resolution occurs. It will be left to the investigator's clinical judgment whether or not an AE is of sufficient severity to require the participant's removal from treatment. A participant may also voluntarily withdraw from treatment due to what he or she perceives as an intolerable AE. If either of these occurs, the participant must undergo an end of study assessment and be given appropriate care under medical supervision until symptoms cease or the condition becomes stable.

The relationship of AEs to the study medication will be assessed by a medically qualified investigator.

### Quality control and quality assurance

To ensure investigators are following the protocol, complying with regulatory and Good Clinical Practice (GCP) standards, and collecting and reporting quality data, sponsors of clinical trials monitor the progress of clinical trials performed by the investigators during the clinical trial. The core components of monitoring are to ensure patient protection and to validate integrity of the data. Monitoring involves periodic on-site visits by monitors each year for the duration of a study as part of a quality process. Significant findings identified as a result of monitoring are escalated for review by the sponsor who may then be managed as a suspected significant deviation. Risk assessments and evaluations are then conducted. There are circumstances where decisions have to be made with regard to taking remedial actions, which may include notifying regulatory authorities and ethics committees of any significant regulatory and/or GCP requirements. At all times, the safety and rights of subjects are the top priority for the trial sponsor.

The clinical trial monitor or clinical research associate (CRA) will verify that the data have been faithfully transcribed from the medical records of patients to the CRF, keeping the confidentiality of patients according to Spanish data protection law. If the CRA detected a mismatch between the CRF data and medical history, he/she will open a query with discordant data to be reviewed by the investigator or investigating staff. This query should be corrected by the investigating team. It is the responsibility of the CRA to check all the open queries in every monitoring visit, to check if it has been corrected and to document the corrections performed.

The documents constituting the master file of the study will include all the documents established in the Good Clinical Practice (CPMP/ICH/135/95). The investigator will warrant that all the people involved in the study will respect the confidentiality of any information on the subjects of the trial, as well as the protection of their personal character data (according to Spanish Law 15/1999, on Protection of Personal Data, from the 13th of December). The anonymity of the participants in the study will be preserved at all times. Only the main researcher of each center and the coordinator of the study will have access to the personal information of participants.

The study data will be transcribed onto CRF and only include a code assigned to each patient, to provide a patient identification code. Only investigators, pharmacists and nurses in the study and authorized by government agencies, if necessary, will be able to access the medical records of patients according to Spanish law 15/1999, on Personal Data Protection, from the 13^th ^of December. This confidential information will remain the exclusive property of the principal investigator and investigating team, may not be disclosed to others without prior written consent from the investigating coordinator and the other principal investigators and may not be used except for this study.

The information originated during the course of this trial is also considered confidential and only to be used by investigators in relation to the objectives of the study and its development.

In all stages of the trial development, patient confidentiality will be guaranteed under current regulations. Once the consent form is signed, a subject code is assigned as detailed in the previous section. This code will be the only possible identification for the patient in the CRF. The CRF consists of AutoCalc paper (original + copy), and will be guarded at all times in a locked place by the researcher responsible for the inclusion of the patient. When the collection of clinical information and follow-up of the subject is finished, the CRA will take the original pages and leave a copy in the custody of the investigator responsible for the inclusion of the patient. Original documents and copies of CRF have to be preserved for at least 10 years from the close-end of the trial. All CRF data on each subject will be turned into a database using SPSS 18.0 version, which is encrypted and stored on external memory devices, in the custody of the CRA and the clinical trial sponsor.

### Ethic, deontological and regulatory considerations

The Investigator will ensure that this study is conducted in accordance with the principles of the Declaration of Helsinki, ICH Guidelines for Good Clinical Practice and in full conformity with relevant regulations.

The protocol, informed consent form, participant information sheet and any applicable documents will be submitted to an appropriate Ethics Committee (EC) and Regulatory Authority for written approval.

All substantial amendments to the original approved documents will also be sent to an appropriate Ethics Committee (EC) and Regulatory Authority for written approval.

The trial staff will ensure that the participants' anonymity is maintained. The participants will be identified only by a participant ID number on the CRF and any electronic database. All documents will be stored securely and only accessible by trial staff and authorized personnel. The study will comply with the Data Protection Legislation which requires data to be anonymized as soon as it is mandatory to do so.

Once it is established that the patient meets the selection criteria for recruitment and before starting treatment, the investigator is responsible for obtaining signed informed consent of patients participating in the study after having provided appropriate information about objectives, methods, anticipated benefits and potential risks. In the event that the patient is unable to read or to give consent, the legal representative must be present during the informed consent process and the legal representative must sign the consent form to attest that the information contained in the form has been explained and understood accurately and, where appropriate, give consent. The investigator must also explain that patients are completely free to refuse to participate in the study or withdraw at any time for any reason.

## Discussion

Preliminary results of perioperative use of EPO with or without intravenous iron in HF are promising [15,16,21]; however, these results suffer from bias because trials were not randomized and/or blinded. With the present CT we are trying to definitively demonstrate the efficacy, and if possible the cost-effectiveness of this clinical practice.

Human recombinant EPO has already demonstrated benefits in cardiac, cancer, orthopedic, and bloodless surgery. With respect to orthopedic surgery, its positive impact is readily apparent in patients undergoing elective procedures associated with substantial blood loss (those with hemoglobin levels > 10 and ≤13 g/dl, whose risk for transfusion is estimated to exceed 10%) [22,23]. Patients undergoing major orthopedic surgery who receive EPO have a nearly six-fold reduction in allogeneic transfusion risk; this benefit extends to elderly patients undergoing major hip or knee reconstruction, and also to more heterogeneous populations of patients needing total joint arthroplasty or major hip or knee surgery [24-28]. These data point towards the hypothesis of similar benefits in the case of HF due to similarities in the diseases, the clinical, anesthetic, and surgical procedures, and postoperative care. Nevertheless, some differences make HF patients a unique population, and results obtained in the previous trials are difficult to extrapolate to patients with HF.

On the other hand, iron deficiency correction by oral iron intake is always slower than the correction obtained with parenteral formulations [29]. There are some retrospective studies showing positive results with the use of iron sucrose in preventing RCP transfusions in patients with HF [14-16]. The authors pointed out that, once injected in vivo, the iron-carbohydrate complexes are metabolized, the iron is released where it then binds transferrin in the plasma, and the redundant carbohydrate moiety is then cleared via the liver [30]. This treatment option increases the erythropoietic effect (4.5-5.5 times that of basal) of intravenous iron, which lasts 7-10 days, after which the iron is sequestered by the reticuloendothelial system [31]. In addition, the availability of iron sucrose, and iron carboxymaltose, two intravenous iron preparations with much lesser and milder side effects than iron dextran, has renewed the interest in these therapies [17-19,32]. In this regard, the effectiveness of iron carboxymaltose, which has additional advantages with respect to sucrose, in the correction of anemia after orthopedic surgery, postpartum, as well as in other medical conditions such as inflammatory bowel disease, heart failure, or chronic kidney disease [17-19,33,34], has prompted us to assess its safety and efficacy in the present trial.

In conclusion we think that this trial will contribute with evidence about the safety and efficacy of ferric carboxymaltose with or without EPO in preventing RPC transfusions in patients with hip fracture.

## PAHFRAC-01 investigators

Máximo Bernabeu-Wittel (1), Mercedes Galván Banqueri (1), Maria Dolores Vega Coca (1), Francisco Javier Galindo Ocaña (1), Ricardo Parra Alcaraz (1), Manuel Rincón Gómez (1), María del Val Martín Sanz (1), Miguel Ángel Giráldez Sánchez (1), Manuel Anaya Rojas (1), Larbi Lezama Núñez (1), Manuel Ollero-Baturone (1), Javier Bautista Paloma (1), Bernardo Santos Ramos (1), Reyes Aparicio Santos (2), Ricardo Espinosa Calleja (2), Boris García Benítez (2), Abelardo Montero Sáez (3), Alberto Ruiz Cantero (4), José Luis Ruiz Arranz (4), José Murcia Zaragoza (5), Javier Santesmases Ejarque (6), Manuel Jesús Romero Jiménez (7), Miguel Ángel Pérez Ramos (7), Miguel Ángel Pascual Díaz (7), María Melero Bascones (8), Teresa Ros Ample (8), Rafael Monte Secades (9), María Guil García (10).

(1) Hospitales Universitarios Virgen del Rocío, Sevilla (HVR).

(2) Hospital San Juan de Dios del Aljarafe, Sevilla (SJD).

(3) Hospital Universitario de Bellvitge, Barcelona (HBV).

(4) Hospital de la Serranía, Ronda, Málaga (HSR).

(5) Hospital de la Vega Baja, Orihuela, Alicante (VBO).

(6) Hospital Germans Trias i Pujol, Badalona, Barcelona (HGT).

(7) Hospital Infanta Elena, Huelva (HIE).

(8) Hospital General Universitario de Albacete (HUA).

(9) Complexo Hospitalario Xeral-Calde, Lugo (HXC).

(10) Hospital Comarcal de la Axarquía. Vélez-Málaga, Málaga (HAX)

## Competing interests

The authors declare that they have no competing interests.

## Authors' contributions

B-WM is promoving the clinical trial, and has wroten the manuscript; RC is the clinical trials' management coordinator of the main center, and has revised the manuscript; ARRM, M-ZJ, M-SR, RC, MA, R-CA, M-BM are the main investigators of the centers, which are leading the inclusion of patients, and have revised the manuscript. All authors read and approved the final manuscript.

## Pre-publication history

The pre-publication history for this paper can be accessed here:

http://www.biomedcentral.com/1471-2474/13/27/prepub
